# Myeloid Neoplasia and Lymphoblastic Lymphoma with Eosinophilia After Radioactive Iodine: A Case Report

**DOI:** 10.4274/balkanmedj.2017.0600

**Published:** 2018-03-15

**Authors:** Mahmut Büyükşimşek, Semra Paydaş, Ali Oğul, Emine Bağır, Melek Ergin

**Affiliations:** 1 Department of Oncology, Çukurova University School of Medicine, Adana, Turkey; 2 Department of Pathology, Çukurova University School of Medicine, Adana, Turkey

**Keywords:** Iodine, lymphoma, myeloid neoplasm, radioactive treatment

## Abstract

**Background:**

Thyroid cancer is the most common endocrine cancer, with an increasing incidence around the world in the last three decades. The increased risk of secondary cancer is associated with a genetic predisposition or radioactive iodine used in the treatment.

**Case Report:**

A 65-year old male patient was operated on for thyroid papillary cancer. He received radioactive iodine on two occasions postoperatively. After six years, he presented with malaise and fatigue with leukocytosis and eosinophlilia. The physical examination revealed inguinal lymphadenopathies and splenomegaly, after examining the bone marrow and lymph node biopsies, he was diagnosed with eosinophilic myeloproliferative neoplasia and T-cell lymphoblastic leukaemia/lymphoma.

**Conclusion:**

Leukaemia and other haematological malignencies may develop after radioactive iodine treatment. Patients with radioactive iodine ablation history should be monitored for a long time.

Thyroid cancer is the most common endocrine cancer, with an increasing incidence around the world in the last three decades (1). Patients can have a long and comfortable life following an appropriate treatment, with a ten-year survival that is expected to be higher than 90% (2). The rate of secondary cancers is known to increase in patients who are diagnosed at a young age and have a long life expectancy. The increased risk of secondary cancer is associated with a genetic predisposition or radioactive iodine (RAI) used in the treatment (3,4,5,6,7).

## CASE PRESENTATION

A 65-year old male was operated upon for thyroid papillary cancer in 2009. Total thyroidectomy was performed. He received RAI twice, with the last one in May 2010. At month 6 following  RAI treatment at a dose of 150 mCi in December 2010, the whole-body scan performed with a 5 mCi dose of I-131 revealed residual thyroid tissue involving the thyroid lodge. The biodistribution of I-131 in the whole body was normal, while pathological radiopharmaceutical accumulation was not detected. The patient was followed up by the endocrinology polyclinic as euthyroid under 150 micrograms levothyroxin treatment, and thyroglobulin levels were not found to be elevated. In June 2011, his white blood cell count was 6.000 Ul (4.3-10.3), haemoglobin was 13.6 g/dL, haematocrit was 40.5% and platelet count was 413.000/μL. The leukocyte count and distribution were normal, while eosinophil count was 12% (0.918 10^3^/microlitre) in December 2012. A slight eosinophil accumulation was observed in his peripheral smear, but no specific cause was found for the eosinophil increase and the patient was followed-up. No abnormality was detected except slight eosinophil increase in the controls performed every 3 months and he was followed up as an asymptomatic patient. The JAK-2 test for MPN was negative.

**Figure 1:**
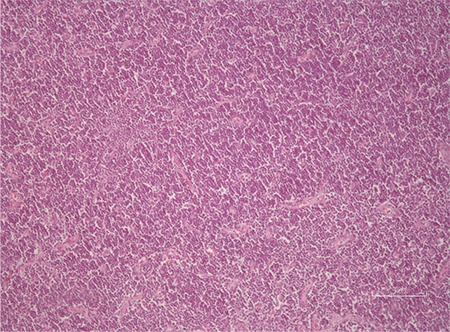
Hypercellular bone marrow biopsy (H&E x200).

**Figure 2:**
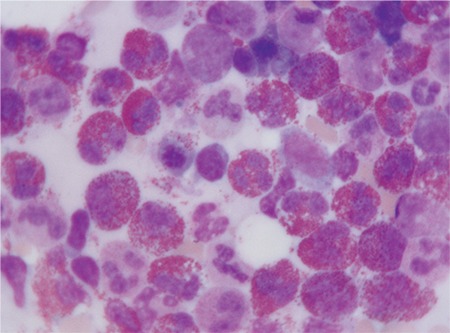
Bone marrow aspiration with prominent eosinophilia (H&E x100).

**Figure 3:**
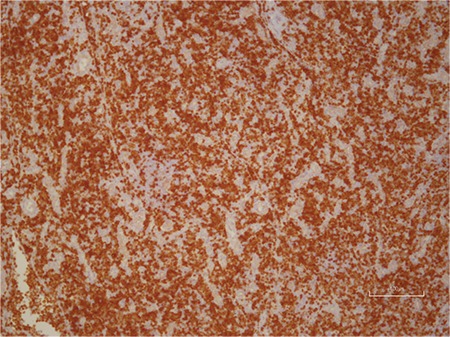
Histological section prepared from the lymph node show diffuse involvement in the paracortical area Tdt x200 by immunohistochemical method.

**Figure 4:**
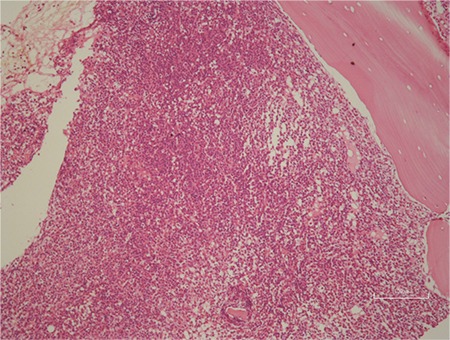
Histological section prepared from the lymph node showing diffuse involvement in the paracortical area (H&E x200).

In May 2016, he presented with malaise and fatigue and was found to have leukocytosis (leucocyte count: 19.600/μL) and anaemia (haemoglobin: 9.5 g/dL). His platelet count was normal while the eosinophil percent was 30.4%. Bone marrow aspiration and biopsy result was interpreted as eosinophilia, myeloid hyperplasia, hypercellular tissue, and myeloproliferative neoplasia (Figure 1, 2) while t(9;22) was negative in his peripheral blood. Tdt, CD34, and CD117 were negative in bone marrow biopsy. Myeloperoxidase was found to be positive in myeloid cells and CD5 was found to be positive in T lymphocytes. Megakaryocytes were found to be increased, but blast cells and mast cells were not increased in bone marrow. The physical examination revealed inguinal lymphadenopathies and splenomegaly. Abdominal ultrasound examination showed that the liver was 18 cm, the parenchymal echo was homogenous and the spleen was 14 cm. Multiple lymph nodes were detected in both inguinal regions, with the most marked one was being 32x13 mm. In June 2016, excisional lymph node biopsy was performed. The report indicated precursor T-cell lymphoblastic leukaemia/lymphoma (Figure 3). Tdt, CD5, CD3, and CD43 were positive, while CD34, CD117, Pax5, Bcl2, CD20, and CD23 were negative (Figure 4). For staging, fluorodeoxyglucose (FDG) positron emission tomography-computed tomography (PET-CT) was performed. His thoracic cross-sections showed multiple lymphadenopathies with minimal F-18 FDG elevation in bilateral axillary regions with the biggest one shown to be around 2.5 cm (SUV_max_: 2.91) by FDG PET-CT and several lymph nodes were found involving minimal F-18 FDG in the mediastinal bilateral hilar regions, with the largest one being around 1 cm (SUV_max_: 2.26); his abdomino-pelvic cross-sections revealed a large spleen size and diffuse increase of F-18 FDG uptake compared to the liver (SUV_max_: 4.08), multiple lymphadenopathies in the bilateral para-aortic, iliac and bilateral inguinal regions (SUV_max_: 7.7) as well as diffuse bone/bone marrow lesions in the entire skeletal system.

*PDGFR-A*, *B* and *FGFR-1* mutation assays were planned; *PDGFR-A* and *B* were found to be negative, while *FGFR-1* was not tested. We used next generation sequencing for *PDGFR-A* and *B* testing. Also, hyperCVAD/MTX+Ara-C chemotherapy was planned for the patient. Metabolic complete response could be achieved after 4 cycles and autologous stem cell transplantation was planned for the patient.

Informed consent has been taken from patient and his relatives.

## DISCUSSION

RAI treatment has a wide application for the treatment of differentiated thyroid cancer. However, the association between secondary cancers and RAI dose is not clear. Two meta-analyses showed that leukaemia, haematological malignancies, salivary gland cancer, colorectal cancer and soft tissue sarcoma might develop after RAI (4). Myeloid and lymphoid neoplasia associated with eosinophilia and specific tyrosine kinase gene fusions have been defined as rare entities. Three cytogenetic abnormalities were described as the fusion of *PDGFR-A*, *PDGFR-B* and *FGFR-1* with other genes. It is known that these genes affect pluripotent stem cells and lead to myeloid neoplasia associated with eosinophilia and rarely T-*lymphoid neoplasia. PDGFR-A* and *PDGFR-B* abnormalities are known to respond well to imatinib, while *FGFR-1* abnormalities are known to be resistant to imatinib and other tyrosine kinase inhibitors. *ETV6-LYN *gene fusion caused by the remodelling between chromosomes 8 and 12 may lead to MPN and T-lymphoblastic lymphoma associated with eosinophilia, while it was included in the eosinophilia and other gene fusion categories by the World Health Organisation. Some patients may benefit from monotherapy with dasatinib or other cytotoxic treatments (5). In one case who entered remission after the treatment of eosinophilia and associated T-lymphoblastic lymphoma and recurrent eosinophilia, complex translocations between chromosomes 7, 12 and 16 were detected, while FISH analysis revealed that the *ETV6* gene (12p13) was responsible for the clonality (6).

Table 1 presents the cases with neoplasia observed and expected after thyroid cancer. A total of 692 cases of second primary malignancy have been identified in 20.235 patients with thyroid cancer. Among them, a total of 11.799 patients (58.3%) received RAI therapy, with median single and cumulated RAI doses of 75 and 100 mCi, respectively (7). Table 2 shows the clinical features of four cases with haematological malignancy after RAI treatment (8). A 45-year old patient followed up for eosinophilia presented with fever and night sweat; his white blood cell count was normal while the peripheral blood had 20% lymphoblasts, and the bone marrow biopsy showed hypercellularity in addition to blasts. *BCR/ABL*, *FIP1L1/PDGFR-A* or *IGH/MYC *fusion genes were found to be negative and the patient was considered to have acute lymphoblastic lymphoma with chronic eosinophilia (9). A thirteen-year old patient presenting with fever and weight loss was considered to have lymphoblastic lymphoma with dominant eosinophilia concordant with FAB2 according to the bone marrow biopsy (10).

We detected eosinophilia in our patient 2 years after 2 cycles of 150 mCi RAI treatment. The patient was followed up as asymptomatic in that period; he became symptomatic 6 years after RAI treatment and myeloproliferative neoplasia. Inguinal lymph node was reported as precursor T-cell lymphoblastic leukaemia/lymphoma. In our patient, *PDGFR-A* and *B* were found to be negative and we could not test for *FGFR-1* and *ETV6-LYN* gene fusion. We know that leukaemia and other haematological malignancies may develop after RAI treatment. However, we present this case because there have been no cases reported in the literature where the patient developed myeloid neoplasia and T-cell lymphoblastic lymphoma associated with eosinophilia after RAI. It should be kept in mind that although these genetic abnormalities and RAI may be a possible cause, the patient may have other genetic risk factors or history accounting for the development of multiple malignancies.

## Figures and Tables

**Table 1:**
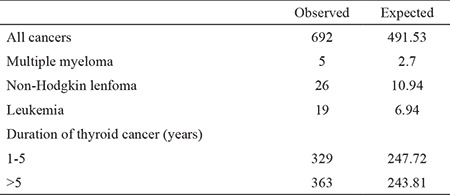
Breakdown of secondary cancers developing after thyroid cancer (14)

**Table 2:**
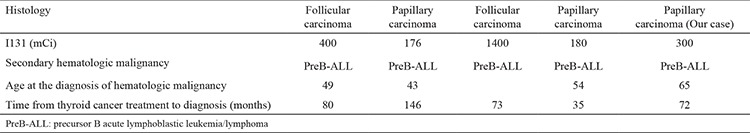
Clinical features of four cases with hematologic malignancy after RAI treatment (15)
